# Combination of Ruxolitinib and Eculizumab for Treatment of Severe SARS-CoV-2-Related Acute Respiratory Distress Syndrome: A Controlled Study

**DOI:** 10.3389/fphar.2020.00857

**Published:** 2020-06-05

**Authors:** Valentina Giudice, Pasquale Pagliano, Alessandro Vatrella, Alfonso Masullo, Sergio Poto, Benedetto Maria Polverino, Renato Gammaldi, Angelantonio Maglio, Carmine Sellitto, Carolina Vitale, Bianca Serio, Bianca Cuffa, Anna Borrelli, Carmine Vecchione, Amelia Filippelli, Carmine Selleri

**Affiliations:** ^1^Department of Medicine, Surgery and Dentistry “Scuola Medica Salernitana” University of Salerno, Baronissi, Italy; ^2^Clinical Pharmacology Unit, University Hospital “San Giovanni di Dio e Ruggi d’Aragona”, Salerno, Italy; ^3^Infectious Disease Unit, University Hospital “San Giovanni di Dio e Ruggi d’Aragona”, Salerno, Italy; ^4^Intensive Care Unit, University Hospital “San Giovanni di Dio e Ruggi d’Aragona”, Salerno, Italy; ^5^Respiratory Disease Unit, University Hospital “San Giovanni di Dio e Ruggi d’Aragona”, Salerno, Italy; ^6^Respiratory Endoscopy Unit, Hospital “Giovanni Da Procida”, University Hospital “San Giovanni di Dio e Ruggi d’Aragona”, Salerno, Italy; ^7^Hematology and Transplant Unit, University Hospital “San Giovanni di Dio e Ruggi d’Aragona”, Salerno, Italy; ^8^Executive Board, University Hospital “San Giovanni di Dio e Ruggi d’Aragona”, Salerno, Italy

**Keywords:** JAK inhibitor, eculizumab, ARDS (acute respiratory distress syndrome), COVID-19, SARS-CoV-2

## Abstract

To date, there are no specific therapeutic strategies for treatment of COVID-19. Based on the hypothesis that complement and coagulation cascades are activated by viral infection, and might trigger an acute respiratory distress syndrome (ARDS), we report clinical outcomes of 17 consecutive cases of SARS-CoV-2-related ARDS treated (N = 7) with the novel combination of ruxolitinib, a JAK1/2 inhibitor, 10 mg/twice daily for 14 days and eculizumab, an anti-C5a complement monoclonal antibody, 900 mg IV/weekly for a maximum of three weeks, or with the best available therapy (N = 10). Patients treated with the combination showed significant improvements in respiratory symptoms and radiographic pulmonary lesions and decrease in circulating D-dimer levels compared to the best available therapy group. Our results support the use of combined ruxolitinib and eculizumab for treatment of severe SARS-CoV-2-related ARDS by simultaneously turning off abnormal innate and adaptive immune responses.

## Introduction

More than 10% of patients with COVID-19 develop severe respiratory symptoms and an acute respiratory distress syndrome (ARDS) requiring intensive care treatment, and precipitating factors are still under investigation; however, a massive release of pro-inflammatory cytokines and chemokines from immune cells is one of the main pathogenetic mechanisms (Channappanavar and Perlman, 2017). The main viral entry mechanism is the binding of the viral “Spike” protein to the surface Angiotensin-Converting Enzyme 2 (ACE-2) receptor on host alveolar epithelial cells (Vaduganathan et al., 2020) likely sequestrating available receptors for renin–angiotensin system regulation and increasing kinin, complement, and coagulation cascade activation. The complement system, a multi-step pathway of the innate immune response, causes pathogen membrane lysis, enhances phagocytosis by macrophages, and releases several pro-inflammatory molecules, such as anaphylatoxins, that amplify inflammation and immune responses. Some of those molecules can trigger the activation of kinin and coagulation cascades which can sustain vascular permeability and thrombosis occurring during ARDS (Gralinski et al., 2018; Campbell and Kahwash, 2020; Risitano et al., 2020), and neutrophil and macrophage recruitment at the site of viral infection supporting inflammation and tissue damage. Simultaneously, infected cells trigger adaptive immune responses, such as Th1 with release of type I interferons, macrophage recruitment and activation with release of IL-1 and TNFα. These mechanisms increase vascular permeability and neutrophil recruitment with production of large amount of pro-inflammatory cytokines, especially IL-6, and anaphylatoxins which sustain inflammation and tissue damage as in a “dog-biting-tail” system until pulmonary edema, thrombosis and respiratory distress develop and progress to ARDS (Cheung et al., 2005). Currently, there are no specific therapies for the COVID-19; however, a lot of available drugs used in other diseases are under investigation also for prophylaxis (Sanders et al., 2020). Based on the hypothesis that SARS-CoV-2 infection triggers a “dog-biting-tail” inflammatory loop, the use of a single anti-inflammatory agent might be not sufficient, thus a combination of drugs would be advisable in preventing ARDS in intensive care patients (Elli et al., 2019; Sanders et al., 2020). In this controlled study, we reported clinical outcomes of severe COVID-19 treated with the novel combination of ruxolitinib and eculizumab which has never been tested before in any disease. The rationale behind this drug combination is that by acting on different but connected pathways triggered by SARS-CoV-2 infection, we can dramatically turn off inflammation in the lungs thus reducing the risk of ARDS.

## Methods

### Patients

A total of 17 consecutive patients were enrolled at the University Hospital “San Giovanni di Dio e Ruggi d’Aragona”, Salerno, Italy, between March 2020 and April 2020 after informed consent obtained in accordance with protocols approved by our local Etic Committee (prot.no. 0052106 on April 1st, 2020; prot.no. 0055788 on April 7, 2020; prot.no. 0057020 on April 9, 2020; and prot.no. 0059417 on April 15, 2020) following current Italian Regulations (law no. D.M. 07.09.2017) for compassionate use of ruxolitinib and eculizumab (**Table 1**). Inclusion criteria were: i) aged 18 or older; ii) diagnosis of SARS-CoV-2 infection confirmed by RT-PCR; and diagnosis of pneumonia or SARS-CoV-2-related ARDS based on the WHO criteria (https://www.who.int/publications-detail/clinical-management-of-severe-acute-respiratory-infection-when-novel-coronavirus-(ncov)-infection-is-suspected). Exclusion criteria were: i) aged 17 or younger; ii) negativity for SARS-CoV-2 infection; iii) diagnosis of mild COVID-19 (Force et al., 2012); iv) active infections including tuberculosis or other clinical conditions that contraindicate the use of eculizumab and/or ruxolitinib; v) hypertransaminasemia and/or cytopenia; vi) pregnant or breastfeeding women. Here, we reported clinical outcomes at day 7 of seven severe COVID-19 patients treated with ruxolitinib and eculizumab, and of 10 severe COVID-19 subjects treated with the best available therapy (BAT group). Safety of combined use of ruxolitinib and eculizumab in patients with COVID-19 was assessed by documenting serious adverse events classified according to the National Cancer Institute, National Cancer Institute Common Terminology Criteria for Adverse Events, version 4.0 (Kluetz et al., 2016). In our cohort, seven out of 17 patients received the combination of ruxolitinib and eculizumab as per following schedule: at day 0, ruxolitinib, 10 mg/twice daily and eculizumab 900 mg IV; ruxolitinib was given twice daily every day for 14 days; while, eculizumab was administered at day 7 and, when needed, at day 14 for a total maximum dose of three. Antibiotic prophylaxis with azithromycin was started in all 17 subjects. Patients on the BAT group (N = 10) did not receive ruxolitinib and eculizumab, while they received subcutaneous heparin (N = 5/10), hydroxychloroquine (HCQ; N = 8/10), and/or mechanical ventilation based on PaO_2_ values. HCQ and subcutaneous heparin were also administered to all subjects in the experimental arm (N = 7), and low-flow oxygen was given when needed based on PaO_2_ values. One patient in the treated arm and two in the BAT group also received an antiviral agent (darunavir/cobicistat or lopinavir/ritonavir). However, because of QT prolongation, antivirals were discontinued in those patients, and not administered to other subjects in this study. Steroids at 20 mg/twice daily were administered in five out of seven subjects in the treated group, and in three out of 10 in BAT group.

**Table 1:** Demographic and Clinical Characteristics of Patients at Baseline.

Characteristics	Ruxolitinib–eculizumab (N = 7)	Best available therapy (N = 10)
Age, median (range)—years	61 (53–70)	63.5 (31–85)
M/F	6/1	7/3
Coexisting conditions		
Arterial hypertension	2/7	7/10
Ischemic cardiopathy	–	3/10
Diabetes	2/7	2/10
Obesity	2/7	2/10
COPD	1/7	2/10
Others		
NHL	1/7	–
β-thalassemia	1/7	–
Alzheimer’s disease	–	1/10
Benign prostatic hyperplasia	–	2/10
Median PaO_2_—mmHg	78 (38–107)	76.5 (55–114)
Median FiO_2_—%	50 (30–60)	21 (21–80)
Median PaO_2_/FiO_2_ mmHg	156 (127–268)	348 (87–505)
PaO_2_/FiO_2_ <300 mmHg	7/7	4/9
Systolic blood pressure		
<90 mmHg	0/7	0/10
>120 mmHg	4/7	8/10
Median WBC (cells/µl)	6,590 (1,210–8,200)	8,610 (6,100–37,660)
Median Lymp (cells/µl)	1,350 (140–7,100)	1,305 (660–2,100)
Median Platelets (×10^3^/µl)	280 (66–454)	237 (118–493)
Median Hemoglobin (g/dl)	12.2 (7.3–15.2)	11 (8.8–17.3)
Median D-dimer (ng/ml)	138 (0.5–2,958)	152 (52–3,464)
Median LDH (IU/L)	656 (297–1,251)	492.5 (347–1,498)
Additional treatments		
Antibiotics	7/7	10/10
Antiviral	1/7	2/10
Low-dose steroids	5/7	3/10
Hydroxychloroquine	7/7	8/10
Heparin	7/7	5/10

COPD, chronic obstructive pulmonary disease; NHL, non-Hodgkin lymphoma; WBC, white blood cells; LDH, lactate dehydrogenase; Lymph, lymphocytes.

### Statistical Analysis

Population size was determined based on the intention-to-treat principle and on the type of study (controlled for compassionate use of drugs) in which a minimum of 12 subjects is required (Moore et al., 2011). All enrolled patients were included in the analysis. Data were analyzed using Prism (v.8.3.0; GraphPad software, La Jolla, CA, USA). Post-hoc analysis included unpaired two-tailed t-tests for two group comparison of clinical parameters and laboratory results at baseline and at day 7. A *P <*0.05 was considered statistically significant.

## Results and Discussion

**Figure 1:**
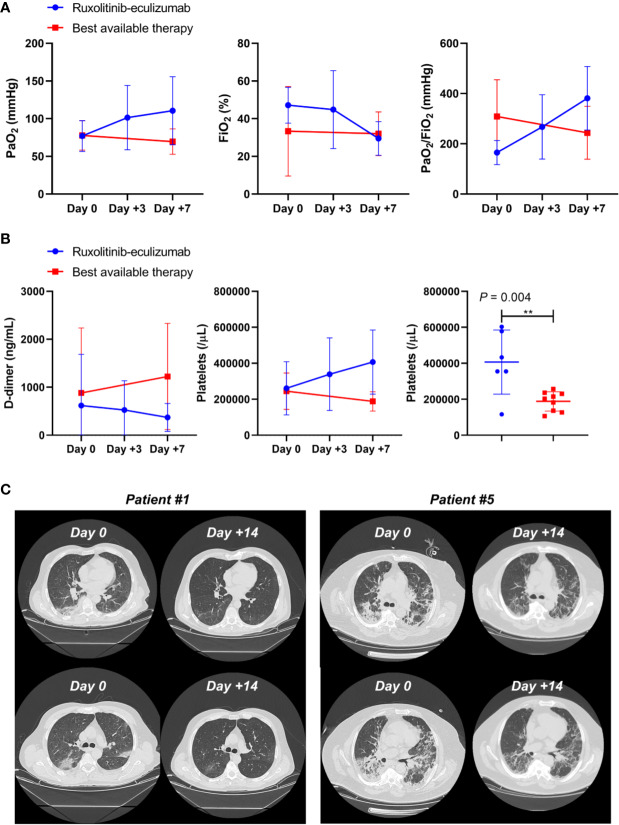
Clinical outcomes in ruxolitinib and eculizumab treated COVID-19 patients. **(A)** PaO_2_, FiO_2_, and PaO_2_/FiO_2_ ratios are reported for ruxolitinib–eculizumab treated (red lines) and best available therapy (blue lines) subjects at days 0, + 3, and +7. Mean ± SD are shown. **(B)** D-dimer levels and platelet counts are displayed for treated (red lines) and best available therapy (blue lines) subjects at days 0, + 3, and +7, and platelet counts are compared by unpaired t-test between groups at day +7. Mean ± SD are shown. **(C)** CT scan imaging at day 0 (Day 0) and after 14 days (Day +14) of ruxolitinib and eculizumab in two representative patients.

Median age of treated patients (N =7) was 61 years, and 86% were male, while the median age of BAT group (N = 10) was 63.5 years and 70% were male. All patients in the treated group had moderate to severe ARDS at admission, while nine out of 10 patients showed moderate to severe ARDS in the BAT group (**Table 1**). At admission, five patients in the treated arm and three in the BAT group received low-flow oxygen, while high-flow oxygen was given to one subject in each arm. Patients on ruxolitinib and eculizumab displayed clinical improvement just after three days of treatment showing higher PaO_2_ and PaO_2_/FiO_2_ ratio, and a decrease in circulating D-dimer levels and an increase in platelet count. At day 7, patients on ruxolitinib and eculizumab displayed a significant improvement in PaO_2_ and PaO_2_/FiO_2_ ratio compared to the BAT group (*P* = 0.0260 and *P* = 0.0395, respectively; unpaired t-test performed) (**Figure 1A**), while no differences were observed for FiO_2_ values (*P* = 0.6630). In addition, subjects on ruxolitinib and eculizumab showed slightly decreased D-dimer levels (*P* = 0.0929), and a significant increase in platelet count compared to BAT group at day 7 (*P* = 0.0038) (**Figure 1B** and **Table 2**). Our findings add additional evidence of the efficacy of eculizumab for treatment of SARS-CoV-2 infection that might trigger an uncontrolled activation of complement and coagulation cascades also causing platelet consumption (Campbell and Kahwash, 2020; Diurno. et al., 2020; Risitano et al., 2020). Evidence shows that the complement factor C5a is directly involved in increasing vascular permeability in pneumococcal meningitis in human and mouse models (Woehrl et al., 2011) thus, by blocking C5a, eculizumab might reduce interstitial damage during SARS-CoV-2-related ARDS and ameliorate oxygen/carbon-dioxide exchanges through respiratory membranes. Moreover, ground-glass unilateral or bilateral opacities were observed for all patients at diagnosis by CT scan imaging, and markedly improvements were documented after 14 days of treatment with ruxolitinib and eculizumab (two representative cases are shown in **Figure 1C**). No differences were described for lactate dehydrogenase levels (*P* = 0.1255) between groups, for hemoglobin levels (*P* = 0.6901), and for white blood cell (*P* = 0.3271) or lymphocyte (*P* = 0.4147; unpaired t-test performed) counts at day 7. In addition, no secondary infections were documented in the treated arm (**Table 2**) suggesting that ruxolitinib 10 mg/twice daily and eculizumab 900 mg IV/weekly for a maximum of three weeks could be not as immunosuppressive as it might be in a long-period treatment, thus this combination might be safely used for treatment of SARS-CoV-2-related ARDS. Indeed, comparable infection rates between ruxolitinib-treated patients and standard-care groups are also reported in myelofibrosis patients from the COMFORT studies, and secondary infections are documented after 12 weeks of treatment with higher dosages (Verstovsek et al., 2012; Cervantes et al., 2013; Verstovsek et al., 2013). Similarly, the risk of meningococcal infection is increased only after several weeks of eculizumab with a meningococcal infection rate of 0.25 per 100 patient/year in pediatric population (Rondeau et al., 2019). In addition, JAK inhibitors have already been largely proposed for treatment of COVID-19 because it might interfere with immune responses triggered by the virus, and several clinical trials are investigating the potential effects of JAK inhibitors as single agent at a daily dose of 10 mg (Galimberti et al., 2020; Matricardi et al., 2020; Seif et al., 2020). One patient with ARDS and pancytopenia on the treated group died on day 5 although an initial clinical improvement. This patient had a clinical history of a stage IVa non-Hodgkin marginal B-cell lymphoma and a hypo-dysplastic bone marrow cellularity, and he was the only subject who received invasive mechanical ventilation at admission among all 17 patients. We also documented an ARDS-related death in one subject of the BAT group at day 7 of hospitalization. We did not register any other severe grade 2 or higher drug-related adverse events, and all remaining patients are alive. However, subjects in the BAT group had higher incidence of lymphopenia (20% *vs* 0%, BAT *vs* treated group), anemia (30% *vs* 14%), and increased creatinine levels (40% *vs* 0%, BAT *vs* treated group) (**Table 2**). In the experimental arm, all subjects stopped ruxolitinib at day +14, while four subjects received three doses of eculizumab and two only two doses at days 0 and 7. In particular, these latter were also discharged at day 16 because of complete clinical recovery, while the other three were discharged at day +28, and one subject was discharged after 44 days of hospitalization because viral RNA was still detectable after ruxolitinib and eculizumab treatment. The median hospitalization time was 24 days (range, 16–44 days) in treated arm, and 34 days (range, 9–60 days) in the BAT group. None of the patients on the treated arm required invasive mechanical ventilation or high-flow nasal oxygenation after or during treatment.

**Table 2:** Clinical outcomes.

Characteristics	Ruxolitinib–eculizumab (N = 7)	Best available therapy (N = 10)	*P* value
Median PaO_2_—mmHg	94 (66–184)	77 (34–87)	***0.0260***
Median FiO_2_—%	27.5 (21–40)	28 (21–50)	0.6630
Median PaO_2_/FiO_2_ mmHg	370.5 (240–594)	246 (114–395)	***0.0395***
Systolic blood pressure			
< 90 mmHg	0/7	0/10	–
>120 mmHg	2/7	2/7	
Median WBC (cells/µl)	8,135 (5,680–10,380)	9,600 (4,900–5,120)	0.3271
Median Lymp (cells/µl)	1,560 (1,130–2,690)	1,415 (370–2,940)	0.4147
Median Platelets (×10^3^/µl)	394 (116–603)	203 (106–257)	***0.0038***
Median Hb (g/dl)	11.95 (9.6–13.5)	11.2 (8.3–15.3)	0.6901
Median D-dimer (ng/ml)	405 (0.27–760)	1073.5 (50–2,680)	0.0929
Median LDH (IU/L)	637.5 (50–729)	346 (158–944)	0.1255
Median hospitalization (days)	24 (16–44)	34 (9–60)	0.3687
Any grade adverse events	N = 13	N = 22	
Lymphopenia	0/7	2/10	
Leukopenia	0/7	0/10	
Thrombocytopenia	0/7	0/10	
Anemia	1/7	3/10	
Increased AST	4/7	4/10	
Increased ALT	5/7	4/10	
Increased total bilirubin	1/7	1/10	
Increased creatinine	0/7	3/10	
ARDS (moderate to severe)	1/7	4/10	
Secondary infections	0/7	0/10	
Death	1/7	1/10	

WBC, white blood cells; Lymph, lymphocytes; Hb, hemoglobin; LDH, lactate dehydrogenase; AST, aspartate aminotransferase; ALT, alanine aminotransferase; ARDS, acute respiratory distress syndrome. P value < 0.05 statistically significant by unpaired t-test and highlighted in bold italic.

In this controlled study, we investigated for the first time ever clinical benefits of a novel combination of ruxolitinib and eculizumab for treatment of an inflammatory life-threatening respiratory distress syndrome triggered by viral infection. Our results showed that this never-tested combination of ruxolitinib and eculizumab could be an alternative for treatment of severe COVID-19 and related ARDS by likely interrupting at multiple steps the inflammatory loop caused by viral infection and immune responses that trigger an uncontrolled activation of kinin, complement, and coagulation cascades (Risitano et al., 2020). Further randomized controlled clinical trials in larger populations are required to confirm our data; however, our preliminary results can open new scenarios for treatment of not only SARS-CoV-2 severe infections, but also of life-threatening conditions, such as ARDS.

## Data Availability Statement

The raw data supporting the conclusions of this article will be made available by the authors, without undue reservation.

## Ethics Statement

The studies involving human participants were reviewed and approved by Comitato Etico Campania Sud. The patients/participants provided their written informed consent to participate in this study.

## Author Contributions

VG and CSelle designed the study. VG, CSelle, PP, AB, CVe, and AF wrote the clinical protocol. PP, AV, AM, AB, SP, BP, RG, AMag, CSelli, CVit BC, and BS enrolled patients and were involved in their clinical managements. BC, AMag and CSelli collected clinical data. VG analyzed the data. VG and CSelle wrote the manuscript. All authors contributed to the article and approved the submitted version.

## Conflict of Interest

The authors declare that the research was conducted in the absence of any commercial or financial relationships that could be construed as a potential conflict of interest.

## References

[B5] Campbell C. M. Kahwash R. (2020). Will Complement Inhibition be the New Target in Treating COVID-19 Related Systemic Thrombosis? Circulation. 10.1161/CIRCULATIONAHA.120.047419 32271624

[B15] Cervantes F. Vannucchi A. M. Kiladjian J. J. Al-Ali H. K. Sirulnik A. Stalbovskaya V (2013). Three-year efficacy, safety, and survival findings from COMFORT-II, a phase 3 study comparing ruxolitinib with best available therapy for myelofibrosis. Blood 122, 4047–4053. 10.1182/blood-2013-02-485888 24174625

[B1] Channappanavar R. Perlman S. (2017). Pathogenic human coronavirus infections: causes and consequences of cytokine storm and immunopathology. Semin. Immunopathol. 39, 529e539. 10.1007/s00281-017-0629-x 28466096 PMC7079893

[B6] Cheung C. Y. Poon L. L. Ng I. H. Luk W. Sia S. F. Wu M. H. (2005). Cytokine responses in severe acute respiratory syndrome coronavirus-infected macrophages in vitro: possible relevance to pathogenesis. J. Virol. 79, 7819–7826. 10.1128/JVI.79.12.7819-7826.2005 15919935 PMC1143636

[B12] Diurno F. Numis F. G. Porta G. Cirillo F. Maddaluno S. Ragozzino A (2020). Eculizumab treatment in patients with COVID-19: preliminary results from real life ASL Napoli 2 Nord experience. Eur. Rev. Med. Pharmacol. Sci. 24, 4040–4047. 10.26355/eurrev_202004_20875 32329881

[B8] Elli E. M. Baratè C. Mendicino F. Palandri F. Palumbo G. A. (2019). Mechanisms Underlying the Anti-inflammatory and Immunosuppressive Activity of Ruxolitinib. Front. Oncol. 9, 1186. 10.3389/fonc.2019.01186 31788449 PMC6854013

[B500] Force A. D. T. Ranieri V. M. Rubenfeld G. D. Thompson B. T. Ferguson N. D. Caldwell E. (2012). Acute respiratory distress syndrome: the Berlin Definition. JAMA. 307 (23), 2526–2533. 10.1001/jama.2012.5669 22797452

[B20] Galimberti S. Baldini C. Baratè C. Ricci F. Balducci S. Grassi S. (2020). The CoV-2 outbreak: how hematologists could help to fight Covid-19. Pharmacol. Res., 104866. 10.1016/j.phrs.2020.104866 32387301 PMC7202852

[B3] Gralinski L. E. Sheahan T. P. Morrison T. E. Menachery V. D. Jensen K. Leist S. R. (2018). Complement Activation Contributes to Severe Acute Respiratory Syndrome Coronavirus Pathogenesis. mBio 9, e01753–e01818. 10.1128/mBio.01753-18 30301856 PMC6178621

[B10] Kluetz P. G. Chingos D. T. Basch E. M. Mitchell S. A. (2016). Patient-Reported Outcomes in Cancer Clinical Trials: Measuring Symptomatic Adverse Events With the National Cancer Institute’s Patient-Reported Outcomes Version of the Common Terminology Criteria for Adverse Events (PRO-CTCAE). Am. Soc. Clin. Oncol. Educ. Book 35, 67–73. 10.14694/EDBK_159514 27249687

[B18] Matricardi P. M. Dal Negro R. W. Nisini R. (2020). The first, holistic immunological model of COVID-19: implications for prevention, diagnosis, and public health measures. Pediatr. Allergy Immunol. 10.1111/pai.13271 PMC7267459 32359201

[B11] Moore C. G. Carter R. E. Nietert P. J. Stewart P. W. (2011). Recommendations for planning pilot studies in clinical and translational research. Clin. Transl. Sci. 4, 332–337. 10.1111/j.1752-8062.2011.00347.x 22029804 PMC3203750

[B4] Risitano A. M. Mastellos D. C. Huber-Lang M. Yancopoulou D. Garlanda C. Ciceri F Lambris J. D (2020). Complement as a target in COVID-19? Nat. Rev. Immunol. 10.1038/s41577-020-0320-7 PMC7187144 32327719

[B17] Rondeau E. Cataland S. R. Al-Dakkak I. Miller B. Webb N. Landau D. (2019). Eculizumab Safety: Five-Year Experience From the Global Atypical Hemolytic Uremic Syndrome Registry. Kidney Int. Rep. 4, 1568–1576. 10.1016/j.ekir.2019.07.016 31890998 PMC6933459

[B7] Sanders J. M. Monogue M. L. Jodlowski T. Z. Cutrell J. B. (2020). Pharmacologic Treatments for Coronavirus Disease 2019 (COVID-19): A Review. JAMA. 10.1001/jama.2020.6019 32282022

[B19] Seif F. Aazami H. Khoshmirsafa M. Kamali M. Mohsenzadegan M. Pornour M. Mansouri D (2020). JAK Inhibition as a New Treatment Strategy for Patients with COVID-19. Int. Arch. Allergy Immunol., 1–9. 10.1159/000508247 PMC7270061 32392562

[B2] Vaduganathan M. Vardeny O. Michel T. McMurray J. Pfeffer M. A. Solomon S. D. (2020). Renin-Angiotensin-Aldosterone System Inhibitors in Patients with Covid-19. N. Engl. J. Med. 382, 1653–1659. 10.1056/NEJMsr2005760 32227760 PMC7121452

[B14] Verstovsek S. Mesa R. A. Gotlib J. Levy R. S. Gupta V. DiPersio J. F (2012). A double-blind, placebo-controlled trial of ruxolitinib for myelofibrosis. N. Engl. J. Med. 366, 799–807. 10.1056/NEJMoa1110557 22375971 PMC4822164

[B16] Verstovsek S. Mesa R. A. Gotlib J. Levy R. S. Gupta V. DiPersio J. F (2013). Efficacy, safety and survival with ruxolitinib in patients with myelofibrosis: results of a median 2-year follow-up of COMFORT-I. Haematologica 98, 1865–1871. 10.3324/haematol.2013.092155 24038026 PMC3856961

[B13] Woehrl B. Brouwer M. C. Murr C. Heckenberg S. G. Baas F. Pfister H. W. (2011). Complement component 5 contributes to poor disease outcome in humans and mice with pneumococcal meningitis. J. Clin. Invest. 121, 3943–3953. 10.1172/JCI57522 21926466 PMC3195471

